# Estimation of US patients with cancer who may respond to cytotoxic chemotherapy

**DOI:** 10.2144/fsoa-2020-0024

**Published:** 2020-05-25

**Authors:** Edward B Maldonado, Scott Parsons, Emerson Y Chen, Alyson Haslam, Vinay Prasad

**Affiliations:** 1 Division of Hematology Oncology, Knight Cancer Institute, Oregon Health & Science University, Portland, OR 97239, USA; 2 Department of Epidemiology & Biostatistics, University of California San Francisco, San Francisco, CA 94143, USA

**Keywords:** chemotherapy, cytotoxic, oncology drugs

## Abstract

**Aims, patients & methods:**

In this retrospective review, we sought to estimate the proportion of patients in the USA with advanced or metastatic cancer who are eligible for and may respond to recommended first-line cytotoxic chemotherapy based on National Comprehensive Cancer Network treatment guidelines.

**Results:**

Among 609,640 patients, we estimate 479,823 (78.7%, 95% CI: 78.6–78.8%) may be eligible for cytotoxic chemotherapy while 189,159 out of 609,640 patients (31.0%, 95% CI: 30.9–31.1%) may have achieved treatment response. The average objective response rate from these regimens was 48.6% (range 9.2 to 90.6%).

**Conclusion:**

Given the large role of cytotoxic agents in cancer, drug development in this space may remain of interest in specific cancer types, and regulatory approval of novel oncology drugs may justify head-to-head comparisons against cytotoxic regimens.

## Lay abstract

This retrospective review sought to estimate patients in the USA with advanced or metastatic cancer who are eligible for and may respond to recommended first-line cytotoxic chemotherapy based on National Comprehensive Cancer Network treatment guidelines. Given our results and the large role of chemotherapy in cancer, drug development in this space may remain of interest in specific cancer types and regulatory approval of novel oncology drugs may justify head-to-head comparisons against cytotoxic regimens.

Cytotoxic cancer medications, which act by interfering with cell division, remain widely utilized either alone or as the backbone for combination therapy in clinical practice. Yet, to date, no empirical analysis has sought to estimate the percentage of cancer patients who could derive response to cytotoxic agents. Notably, such an analysis has been performed for nonradiologic, novel genome-targeted therapies [1], and more recently immunotherapy checkpoint inhibitors [2,3].

For this reason, we sought to quantify the contribution of cytotoxic chemotherapy in cancer treatment in 2018 by estimating the percentage of US patients with metastatic cancer who would be eligible for and would respond to cytotoxic chemotherapy alone. In doing so, we provide historical context to the promise of diverse drug development in oncology today.

## Materials & methods

### Overview

We sought to estimate the percentage of patients with advanced or metastatic cancer in 2018 who were eligible for and may have responded to National Comprehensive Cancer Network (NCCN) recommended ‘preferred’ (category 1 and 2A) cytotoxic chemotherapy regimens. We defined eligible for as having a diagnosis for which cytotoxic therapies were advised and defined respond to according to available published sources to estimate what proportion of patients might expect to experience a treatment response (complete response + partial response) to therapy if all were able to be treated. This optimistic assumption was also used in our prior work evaluating the potential impact of genome directed agents [1] and immunotherapy [2]. Our study was not submitted for institutional review board approval, as all of the data were publicly available without protected health information. The study was conducted between 1 October 2018 and 31 December 2018.

Furthermore, in order to estimate the proportion of patients who were eligible for cytotoxic therapy, we first looked at all of the NCCN guidelines for all types of cancer, as of 15 October 2018 [4]. We then modeled Siegel *et al.’*’s classification of the types of cancer, to create our list of cancers included in the study (based on the correlated NCCN guideline by cancer site available) [4,5]. This list of cancers included are also outlined in Table 1 & Supplementary Tables. Ultimately, we operate under the assumption that the estimated number of deaths included in the American Cancer Society (ACS) data would be a surrogate of those who would have been presented with advanced or metastatic cancer. Please refer to Supplementary Table 2 for complete list of excluded cancer types with their rationales. Some specific examples of exclusion criteria include those with ACS-undefined cancers (e.g., ‘other’ digestive organs), those whose mainstay of cancer management includes surgery, exclusion of response data in regions other than the neoadjuvant setting, exclusion of response rates for chemotherapy with concurrent radiation. Additionally, certain types of cancers were excluded altogether due to a variety of reasons including use of radiation, surgery or use of endocrinologic or genome-derived therapies. At last, some cancers were excluded because there was lack of an associated NCCN-guideline for that specific cancer (e.g., small intestine).

**Table 1.** List of cancer types, number of cytotoxic chemotherapy regimens and sample regimen.

Cancer type	Number of regimens	Example regimen
Head and neck cancer (squamous cell carcinoma)	5	Cisplatin/fluorouracil
Esophageal and esophagogastric junction cancer (squamous cell carcinoma & adenocarcinoma)	4	Fluorouracil/oxaliplatin
Gastric cancer	4	Fluorouracil/oxaliplatin
Colorectal cancer	5	Fluorouracil/oxaliplatin/leucovorin
Anal carcinoma	2	Cisplatin/fluorouracil
Biliary tract cancers (gallbladder, intrahepatic/extrahepatic cholangiocarcinoma)	1	Gemcitabine/cisplatin
Pancreatic adenocarcinoma	4	Gemcitabine/cisplatin
Lung cancer	17	Carboplatin/etoposide
Osteosarcoma	2	Cisplatin/doxorubicin
Soft tissue sarcomas	7	Gemcitabine/docetaxel
Breast cancer	9	Doxorubicin
Cervical cancer	3	Cisplatin/paclitaxel
Uterine cancer	1	Carboplatin/paclitaxel
Ovarian cancer (epithelial, fallopian tube & peritoneal)	2	Carboplatin/gemcitabine
Vulvar cancer (squamous cell carcinoma)	1	Cisplatin/fluorouracil
Prostate cancer	1	Docetaxel
Testicular cancer (germ cell tumors)	3	Bleomycin/etoposide/cisplatin
Penile cancer	1	Cisplatin/ifosfamide/paclitaxel
Bladder (urothelial) cancer	3	Gemcitabine/cisplatin
Central nervous system cancers	2	Temozolomide
Hodgkin lymphoma	2	Doxorubicin/bleomycin/vinblastine/dacarbazine
Non-Hodgkin lymphoma	3	Cyclophosphamide/daunorubicin/vincristine/prednisone
Acute lymphoblastic leukemia	3	Daunorubicin/vincristine/prednisone/pegaspargase
Chronic lymphocytic leukemia/small lymphocytic leukemia	1	Fludarabine/cyclophosphamide
Acute myeloid leukemia	2	Daunorubicin/cytarabine

### Data selection

We used annual mortality statistics by cancer type from the ACS from 2018 to obtain the estimated number of patients who died annually from an indication for which there was a recommended cytotoxic chemotherapy regimen (not taking into account functional assessments) [5]. We used death from cancer to estimate those who would have presented with advanced or metastatic cancer. Of note, laryngeal cancer data were grouped with cancer of the oral cavity and pharynx and classified as head and neck cancer. Likewise, intrahepatic/extrahepatic cholangiocarcinoma and gallbladder cancers were collectively classified as biliary tract cancers.

We identified all NCCN-recommended ‘preferred’ (category 1 and 2A) chemotherapy regimens for metastatic disease based on the most up-to-date NCCN guidelines as of 15 October 2018 via the NCCN website [4].

### Data extracted

We then examined the primary source from each NCCN cytotoxic chemotherapy regimen in order to extract the response rate (complete response plus partial response) for that indication.

For combination therapy that included either genome-derived therapy or immunotherapy, we used the reported response rate in the control arms (with only cytotoxic chemotherapy agents). For example, in colorectal cancer, several clinical trials utilized cytotoxic chemotherapy in conjunction with bevacizumab; for those we only utilized data from the control arm (cytotoxic chemotherapy alone). Additionally, for those clinical trials that included radiation therapy, response rates were obtained for chemotherapy agents prior to receiving radiation. Cytotoxic chemotherapy with concurrent radiation were excluded.

A number of cancer types had specifically defined rules in our estimation of patient eligibility. First, cancers where there were no NCCN-recommended cytotoxic chemotherapy regimens (i.e., small bowel cancers) were deemed as not cytotoxic-eligible, therefore no response rate could be calculated. Second, cancers treated with primarily noncytotoxic agents (i.e., multiple myeloma) were also deemed as not cytotoxic-eligible.

Similarly, a number of cancer types had specifically defined rules in our estimation of treatment response. For soft tissue sarcomas, osteosarcomas, vulvar cancer and penile cancers, standard chemotherapy regimens used in the metastatic setting were established on the basis of surgical pathologic response in the perioperative setting; so this response rate end point was used in our estimation of treatment response. For central nervous system (CNS) cancers like glioblastomas, response rate was estimated from second-line chemotherapy because both surgery and definitive chemo-radiation were universally used in the first-line setting. With regards to aggressive lymphomas and acute leukemias, only complete remission (not partial response) was used to estimate response, as partial response may not be clinically meaningful with these diagnoses.

Each of the clinical trials were independently reviewed by three researchers (E Maldonado, S Parsons & EY Chen) to ensure the correct response rates for each cancer.

### Estimating the number of patients who respond to cytotoxics

After obtaining the response rates from the primary literature, the mean (average) of response rate (complete response + partial response for most cancer types) was calculated for each cancer. We then multiplied the estimated mortality in 2018 for that particular cancer site by the averaged response rate. For example, for head and neck cancers, the average response rate of all the cytotoxic chemotherapy regimens was 43.2%. The total estimated number of cancer deaths due to head and neck cancers was 13,740 (men and women). Thus, the estimated proportion of patients who would respond to cytotoxic chemotherapy was calculated to be 5936 out of 609,640 total patients who died from cancer, or 0.97% could respond to cytotoxic chemotherapy for head and neck cancers. This calculation was applied to all of the listed cancer types (Table 2). This is similar to our prior work.

**Table 2.** Average response rate and percentage of treatment response by cancer type.

Cancer type	Average response rate	Number of deaths in 2018	Number of patients benefiting from chemotherapy	Percentage of total patients responding to chemotherapy
Head and neck cancer (squamous cell carcinoma)	43.20%	13,740	5936	0.97
Esophageal and esophagogastric junction cancer (squamous cell carcinoma & adenocarcinoma)	33.20%	15,850	5262	0.86
Gastric cancer	33.20%	10,800	3586	0.59
Colorectal cancer	45.70%	50,630	23,138	3.8
Anal carcinoma	54.40%	1160	631	0.1
Biliary tract cancers (gallbladder, intrahepatic/extrahepatic cholangiocarcinoma)	26.10%	7483	1953	0.32
Pancreatic adenocarcinoma	20.40%	44,330	9057	1.49
Lung cancer	39.10%	154,050	60,234	9.88
– Small cell carcinoma (12.9%)		19,872	7770	1.27
– Squamous cell carcinoma (23.2%)		35,740	13,974	2.29
– Nonsquamous cell carcinoma (63.9%)		98,438	38,489	6.31
Osteosarcoma	50.40%	1590	801	0.13
Soft tissue sarcomas	24.50%	5150	1262	0.21
Breast cancer	34.60%	41,400	14,324	2.35
Cervical cancer	42.20%	4170	1760	0.29
Uterine cancer	75.00%	11,350	8513	1.4
Ovarian cancer (epithelial, fallopian tube & peritoneal)	57.40%	14,070	8076	1.32
Vulvar cancer (squamous cell carcinoma)	60.00%	1200	720	0.12
Prostate cancer	14.50%	29,430	4267	0.7
Testicular cancer (germ cell tumors)	73.70%	400	295	0.05
Penile cancer	50.00%	380	190	0.03
Bladder (urothelial) cancer	54.50%	18,200	9919	1.63
Central nervous system cancers	9.20%	16,830	1548	0.25
Hodgkin lymphoma	74.60%	1050	783	0.13
Non-Hodgkin lymphoma	74.20%	19,910	14,773	2.42
Acute lymphoblastic leukemia	90.60%	1470	1332	0.22
Chronic lymphocytic leukemia/small lymphocytic leukemia	58.00%	4510	2616	0.43
Acute myeloid leukemia	76.70%	10,670	8184	1.34

For majority of the cancers included in the study, primary literature used a combination of the response evaluation criteria in solid tumours (RECIST) and WHO criteria to evaluate response. However, for certain regimens within a specific cancer, Southwest Oncology Group (SWOG) standard response criteria was utilized. Additionally, osteosarcoma response was based on histologic response criteria. Hodgkin lymphoma response was evaluated by International Harmonisation Criteria applied to PET scan. Non-Hodgkin lymphoma response as based on International Workshop Criteria. Acute lymphoblastic leukemia was reported as a ‘hematologic’ response as defined by authors. ‘Chronic lymphocytic leukemia/small lymphocytic leukemia’ was defined by National Cancer Institute Working Group 1996 criteria. At last, ‘acute myeloid leukemia’ response was defined via bone marrow biopsy response.

### Statistical analysis & figure representation

We sought to provide a descriptive estimate of percentage of US patients with metastatic cancer who responded to cytotoxic chemotherapy regimens. Analysis was performed using Microsoft Excel and R, version 3.4.4. Figures were created using Microsoft Excel and were modeled after the figures used by our colleagues on a prior publication evaluating genome-targeted therapies [1].

## Results

We examined a total of 88 different cytotoxic chemotherapy regimens used in 25 different types of cancer. Table 1 lists the number of regimens studied for each cancer type along with representative regimens. Additionally, please refer to Supplementary Table 1 for a full list of regimens studied along with the corresponding clinical trials from which a response rate was collected.

### Patients eligible for cytotoxic chemotherapy

We estimated that in 2018, 479,823 out of a total 609,640 patients (78.7%, 95% CI: 78.6–78.8%) were eligible for the cytotoxic chemotherapy regimens recommended by the NCCN. The cancers that accounted for the greatest percentage of total deaths for which patients were not eligible for cytotoxic chemotherapy are hepatocellular carcinoma (n = 21,593; 3.54%), nonbasal cell or squamous cell skin cancers (n = 13,460; 2.21%), as well as cancers falling under the category ‘other or unspecified primary site’ (n = 44,560; 7.31%).

### Patients responding to cytotoxic chemotherapy

**Figure 1.**
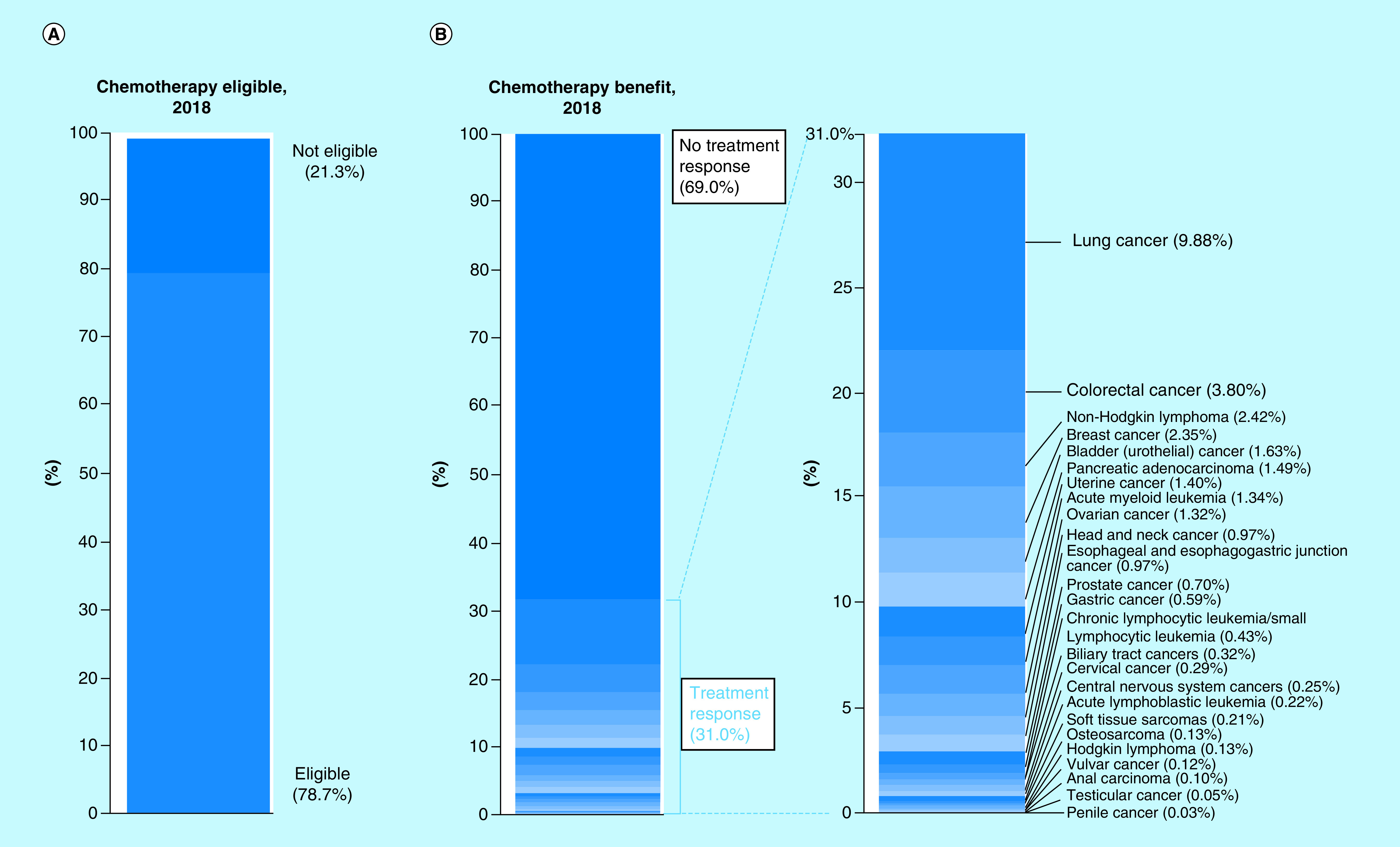
Estimation of US patients who are eligible for and may respond to cytotoxic chemotherapy, 2018.

**Figure 2.**
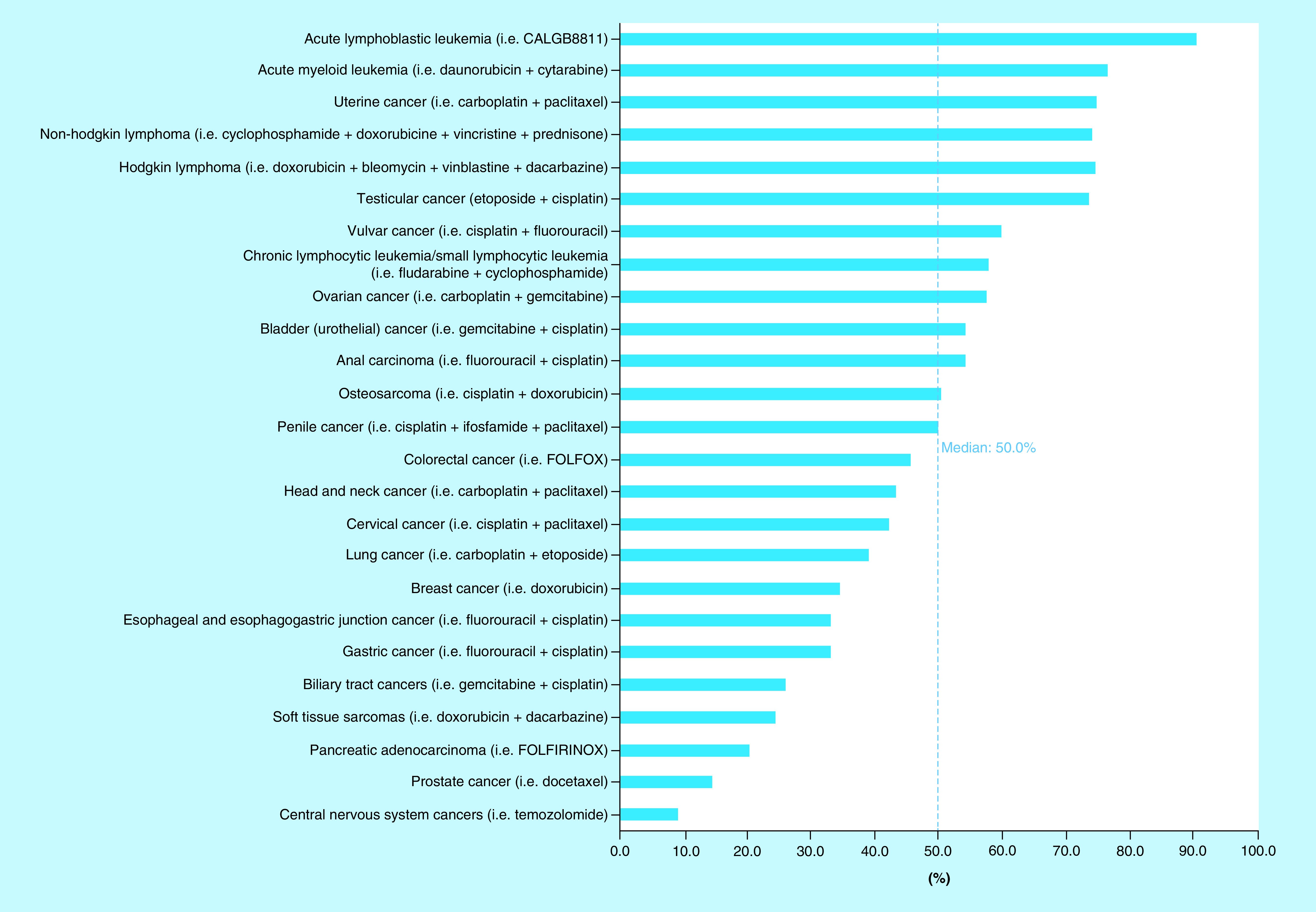
Estimated average response rates for cytotoxic chemotherapy regimens, 2018.

The estimated number of patients in 2018 who were both eligible for and would respond to cytotoxic NCCN-recommended ‘preferred’ (category 1 and 2A) chemotherapy regimens was 189,159 out of 609,640 total cancer deaths, or 31.0% (95% CI: 30.9–31.1%; Figure 1). The estimated number of patients who would not respond to cytotoxic chemotherapy was 420,481 out of 609,641 total cancer deaths, or 69.0%. Out of the 69.0% of patients who were estimated to not achieve at least partial response from cytotoxic chemotherapy, 21.3% of those have a cancer that is not eligible for cytotoxic chemotherapy as first-line treatment. Thus, the estimated number of patients who are eligible for cytotoxic therapy but would not achieve a response was 290,664 of out 609,640 or 47.7% (95% CI: 47.6–47.8%). Table 2 lists the average response rate (not weighed by frequency of tumor type) as well as the estimated number of patients who would respond to cytotoxic chemotherapy for each cancer type. The average response rate for all cancers included in our study was 48.6% (95% CI: 40.4–56.9%), with a median response rate of 50% (Figure 2). Cancers with the highest average response rate included acute lymphoblastic leukemia (n = 1332; 90.6%), acute myeloid leukemia (n = 8184; 76.7%) and uterine cancer (n = 8513; 75%) while the cancers with the lowest average response rate were CNS cancers (n = 1548; 9.2%) and prostate cancer (n = 4267; 14.2%). With regards to the cancers in which the greatest total number of patients would respond to cytotoxic chemotherapy, lung cancer (n = 60,234; 9.88% of total number of patients), colorectal cancer (n = 23,138; 3.80%) and non-Hodgkin lymphoma (n = 14,773; 2.42%) were among the top.

## Discussion

Our results suggest the percentage of patients eligible to receive cytotoxic chemotherapy for metastatic malignancies remains high (i.e., 78.7%); this figure can be examined alongside prior reports using identical methodology which found that 8.33% of cancer patients were eligible for genome-targeted therapies [1]. Among all cancer patients, approximately 31.0% may achieve either a partial or complete response to cytotoxic chemotherapy; this again may be viewed alongside a comparable study of genome-targeted agents (4.90%) [1]. With increased use of immunotherapeutics, a recent analysis of US patients with cancers showed approximately 10–9–11.4% possible response to immunotherapeutics alone [2,3].

Drug development in cytotoxic chemotherapy has slowed given enthusiasm for genomic and immunotherapeutic approaches to cancer. For instance, among US FDA approved cancer drugs since 2000, 65 were targeted, compared with only 13 cytotoxic agents [6]. There have been significant advances regarding cancer treatments as of late, including but not limited to earlier detection of primary tumors, increased availability and accessibility of genomic testing; increased utilization of more novel genomic and immunotherapeutic agents (e.g., use of imatinib in chronic myeloid leukemia and use of BRAF inhibitors in melanoma). However, there have not been significant advancements in chemotherapeutics for later stage/metastatic disease, also outlined in our particular study. One study looking at FDA-approval of anticancer agents from 1949 to 2014 showed majority of cytotoxic agents being approved as early as 1941, but really increasing between 1991 and 2000 with the additional approval of 21 cytotoxic agents. However, between 2011 and 2014, only three cytotoxic agents were approved compared with 33 targeted agents [6]. Given that genome-targeted agents are often only applicable to a subset of cancer patients with unique biomarkers, and given that immunotherapy responses remain partly unpredictable, the development and optimization of cytotoxic regimens, which often have little restriction based on molecular biomarkers and result in tumor shrinkage in many patients, remain important.

While the oncology community is aware of the potential and, at times, devastating side effects of cytotoxic chemotherapy regimens, all novel agents carry risks. For example, immunotherapy toxicities include dermatitis, pruritus, hypophysitis, pneumonitis, sarcoidosis, inflammatory arthritis, infusion reactions, cardiac toxicities, various cytopenias, colitis/hepatitis, nephritis, encephalopathy (including leukoencephalopathy and posterior reversible leukoencephalopathy syndrome), peripheral motor and sensory neuropathies, and uveitis/episcleritis (that can result in blindness) [7–9]. While many targeted agents have favorable side effect profiles, there is a growing concern regarding the cardiovascular toxicities, including hypertension, peripheral vascular disease and even atherosclerotic or thrombotic events [10]. Depending on the severity of these toxicities, patients can undergo significant morbidity and potentially life-threatening complications. Thus, all types of cancer therapies incur toxicities.

## Limitations

Our study has several limitations. First, ours is merely an estimate of the use of these drugs and their objective response rate. The actual use and benefit would rely on performance status for potential eligibility, other comorbidities/lab abnormalities that may preclude specific cytotoxic agents within a regimen, clinical circumstances, cost prohibitive circumstances or patient/familial values (i.e., patients may choose not to pursue chemotherapy due to potential morbidity or desire to pursue a more comfort-centered approach). However, while there is cost associated with cytotoxic agents, noncytotoxic agents and more novel therapies are often just as costly, if not more so. It is also important to note that objective tumor response rates in the clinical trials are often much higher than those observed in clinical practice. However, use of objective tumor response alone likely underestimates potential patient benefit, as use of cytotoxic agents may help provide symptomatic improvement and potential changes in progression-free survival or overall survival, even without an objective tumor response.

While cytotoxic agents still play an important role in the future of cancer management, it is important to advocate for the broader optimization of therapies, including combined regimens utilizing a variety of cytotoxic and noncytotoxic agents. Of course, prior Phase I trials have shown low overall response rates when testing mainly cytotoxic agents, ranging from 4 to 6% [11]. Also, there is a dose-dependent association of cytotoxic agents and tumor response, but at the expense of increased toxicity [11]. Interestingly, there have been increased interest in utilization of cytotoxic agents with some novel targeted agents or even immunotherapeutics, with some thought about certain cytotoxic agents possibly having an effect on sensitizing cancer cells to these therapies, as has been demonstrated with PD-L1 inhibitors [12].

There were several cancers that were excluded from the data analysis due to a variety of reasons including, but not limited to, concurrent radiation therapy, prior surgery, use of targeted therapies/immunotherapies/endocrinologic therapies or rarity of disease (refer to Materials & Methods section above & Supplementary Table 2 for further details). Additionally, while many cancer settings utilize specific antibodies in conjunction with cytotoxic chemotherapy regimens, our results regarding average response rates are likely to be lower than the current standard of care for those specific cancer types, as we excluded the additive response rate of these drugs. Some of the preferred chemotherapy regimens had corresponding primary clinical trials without a control arm, without any noncytotoxic chemotherapy agents. Also, some of the corresponding primary clinical trials did not include response rate as an end point, thus leading to omission of the data from that specific trial. All of these exclusions would only lower the reported estimates.

Second, annual deaths from a specific cancer were used as a surrogate for the number of patients with advanced or metastatic cancer in order to estimate the eligibility and benefit of cytotoxic chemotherapies for the specific malignancies included in this study. By utilizing mortality data, our calculations for eligibility and response to cytotoxic chemotherapy regimens during the year 2018 includes malignancies that were actually diagnosed in prior years. However, given the conservative assumptions made during the data collection (i.e., excluding partial responses for various hematologic malignancies including but not limited to Hodgkin lymphoma, non-Hodgkin lymphoma, etc.), our estimates possibly represent an upper boundary.

It is also important to note that many of the clinical trials from which response rates were obtained do not provide adequate subgroup analysis regarding specific racial/ethnic minorities; thus cannot be generally applied to those from more diverse backgrounds. While we did not aim to identify the implications of cytotoxic agents in specific subgroups of the population, it raises a larger issue of the need for future clinical trials to take into account the broader applicability of these cancer agents within our more interconnected and increasingly diverse patient populations to help address important healthcare disparities permeating every area of medical care.

Third, it is possible that some patients who may have had stable disease could have benefitted cytotoxic chemotherapy. Yet, exclusion of stable disease rates was consistent with prior work and stable disease is a broad category that includes slowly growing tumors that have not met Response Evaluation Criteria in Solid Tumours or WHO progression threshold.

## Conclusion & future perspective

Despite increased interest in genome-targeted therapies and immunotherapy, cytotoxic chemotherapy retains widespread importance in cancer medicine. We estimate that a large proportion of US patients may potentially be eligible for and respond to cytotoxic chemotherapy. We encourage others to build on our findings with more rigorous analysis of the potential benefits of utilizing cytotoxic agents. Alongside investments in novel drug discovery, we favor that some portfolio of cancer drug development continues to research and optimize cytotoxic chemotherapy. Further prospective studies should be aimed at identifying how cytotoxic chemotherapy-associated objective response correlates with progression-free survival and overall survival in this setting. Additionally, we advocate for continued optimization of cancer treatment regimens, with various combinations of cytotoxic agents, in conjunction with targeted therapies and immunotherapeutics. At last, with increasingly more diverse and interconnected patient populations, trialists should begin to increase data reporting specific to racial/ethnic subgroup analyses to help with broad applicability of treatment recommendations.

**Summary points**

- Question: How many US patients with advanced or metastatic cancer are eligible for and may respond to standard first-line cytotoxic chemotherapy regimens?
- Findings: Among an estimated 609,640 cancer-related deaths in 2018, 479,823 (78.7%) would have been eligible to receive standard first-line cytotoxic chemotherapy, and 189,159 (31.0%) would have been expected to achieve treatment response.
- Meaning: Cytotoxic agents remain the important treatment options for patients in the USA with cancer.
