# Supplementary material for: Estimation of US patients with cancer who may respond to cytotoxic chemotherapy
Source: Future Sci OA. 2020 May 25;6(8):FSO600. doi: 10.2144/fsoa-2020-0024 (PMC7491026; doi:10.2144/fsoa-2020-0024)
Supplement: Supplementary file 1 [file fsoa-06-600-s1.docx]

**Supplemental Table 1. Complete List of Cytotoxic Chemotherapy Regimens and Response Rate by Cancer Type**.^a^

| Cancer Type | Chemotherapy Regimen | Overall Response Rate (%) | Complete Response (%) | Partial Response (%) |
| --- | --- | --- | --- | --- |
| Head and Neck Cancer (Squamous Cell Carcinoma) | Cisplatin or Carboplatin/Docetaxel | 20.0% | - | - |
|  | Cisplatin or Carboplatin/Docetaxel | 76.5% | 17.6% | 58.9% |
|  | Cisplatin or Carboplatin/Docetaxel | 25.0% | 7.0% | 18.0% |
|  | Cisplatin or Carboplatin/Paclitaxel | 26.0% | 7.0% | 19.0% |
|  | Cisplatin or Carboplatin/Paclitaxel | 61.7% | 4.2% | 57.5% |
|  | Cisplatin/Placebo | 9.8% | - | - |
|  | Cisplatin/Fluorouracil | 29.8% | 6.7% | 23.1% |
|  | Cisplatin/Fluorouracil | 32.0% | 6.0% | 26.0% |
|  | Cisplatin/Fluorouracil | 60.2% | 2.8% | 57.4% |
|  | Cisplatin/Fluorouracil | 42.0% | 2.8% | 39.2% |
|  | Cisplatin/Gemcitabine | 71.1% | 6.9% | 64.2% |
|  | Cisplatin/Gemcitabine | 64.1% | 8.3% | 55.8% |
| Esophageal and Esophagogastric Junction Cancer (Squamous Cell Carcinoma & Adenocarcinoma) | Fluorouracil/Cisplatin | 34.0% | 2.0% | 32.0% |
|  | Fluorouracil/Cisplatin | 30.0% | 3.0% | 27.0% |
|  | Fluorouracil/Cisplatin | 16.7% | - | - |
|  | Fluorouracil/Cisplatin | 27.0% | 0.0% | 27.0% |
|  | Capecitabine/Cisplatin | 46.0% | 2.0% | 44.0% |
|  | Fluorouracil/Oxaliplatin | 34.8% | 3.6% | 31.2% |
|  | Capecitabine/Oxaliplatin | 44.0% | 2.0% | 42.0% |
| Gastric Cancer | Fluorouracil/Cisplatin | 34.0% | 2.0% | 32.0% |
|  | Fluorouracil/Cisplatin | 30.0% | 3.0% | 27.0% |
|  | Fluorouracil/Cisplatin | 16.7% | - | - |
|  | Fluorouracil/Cisplatin | 27.0% | 0.0% | 27.0% |
|  | Capecitabine/Cisplatin | 46.0% | 2.0% | 44.0% |
|  | Fluorouracil/Oxaliplatin | 34.8% | 3.6% | 31.2% |
|  | Capecitabine/Oxaliplatin | 44.0% | 2.0% | 42.0% |
| Colorectal Cancer | FOLFOX | 50.7% | - | - |
|  | FOLFOX | 72.0% | 4.0% | 68.0% |
|  | FOLFOX | 39.0% | - | - |
|  | FOLFOX (RR for WT-KRAS) | 48.0% | - | - |
|  | FOLFOX (RR for MT-KRAS) | 40.0% | - | - |
|  | Capecitabine/Oxaliplatin | 38.0% | - | - |
|  | FOLFIRI | 47.2% | 5.3% | 41.9% |
|  | FOLFOXIRI | 66.0% | 8.0% | 58.0% |
|  | Capecitabine | 10.0% | 1.0% | 9.0% |
| Anal Carcinoma | Fluorouracil/Cisplatin | 63.0% | 9.0% | 54.0% |
|  | Fluorouracil/Cisplatin | 63.2% | 5.3% | 57.9% |
|  | Carboplatin/Paclitaxel | 37.0% | 4.0% | 33.0% |
| Biliary Tract Cancers (Gallbladder, Intrahepatic / Extrahepatic Cholangiocarcinoma) | Gemcitabine/Cisplatin | 26.1% | - | - |
| Pancreatic Adenocarcinoma | FOLFIRINOX | 31.6% | 0.6% | 31.0% |
|  | Gemcitabine/Albumin-Bound Paclitaxel | 22.9% | 0.2% | 22.7% |
|  | Gemcitabine | 8.0% | - | - |
|  | Gemcitabine/Cisplatin | 19.1% | 3.0% | 16.1% |
| Lung Cancer: SCLC | Carboplatin/Etoposide | 64.4% | 1.0% | 63.4% |
|  | Carboplatin/Etoposide | 75.0% | - | - |
|  | Carboplatin/Etoposide | 48.0% | 0.0% | 48.0% |
|  | Cisplatin/Etoposide | 86.0% | 43.0% | 43.0% |
|  | Cisplatin/Etoposide | 65.0% | 10.0% | 55.0% |
|  | Carboplatin/Irinotecan | 67.0% | 12.0% | 55.0% |
|  | Cisplatin / Irinotecan | 84.4% | 2.6% | 81.8% |
|  | Cisplatin / Irinotecan | 48.0% | - | - |
| Lung Cancer: NSCLC | Carboplatin/Albumin-Bound Paclitaxel | 41.0% | - | - |
|  | Carboplatin/Albumin-Bound Paclitaxel | 33.0% | 0.0% | 33.0% |
|  | Carboplatin/Albumin-Bound Paclitaxel | 38.4% | 2.1% | 36.3% |
|  | Carboplatin/Docetaxel | 23.9% | 1.2% | 22.7% |
|  | Carboplatin/Etoposide | 16.0% | 1.0% | 15.0% |
|  | Carboplatin/Etoposide | 21.7% | - | - |
|  | Carboplatin/Gemcitabine | 30.0% | 1.0% | 29.0% |
|  | Carboplatin/Paclitaxel | 32.4% | - | - |
|  | Carboplatin/Paclitaxel | 38.4% | 2.1% | 36.3% |
|  | Carboplatin/Pemetrexed | 31.6% | 0.0% | 31.6% |
|  | Carboplatin/Pemetrexed | 29.0% | 0.0% | 29.0% |
|  | Carboplatin/Pemetrexed | 18.9% | - | - |
|  | Cisplatin/Docetaxel | 31.7% | 2.0% | 29.7% |
|  | Cisplatin/Etoposide | 21.9% | 0.0% | 21.9% |
|  | Cisplatin/Gemcitabine | 30.1% | - | - |
|  | Cisplatin/Gemcitabine | 28.2% | - | - |
|  | Cisplatin/Paclitaxel | 21.0% | 0.0% | 21.0% |
|  | Cisplatin/Pemetrexed | 30.6% | - | - |
|  | Cisplatin/Pemetrexed | 18.9% | - | - |
|  | Gemcitabine/Docetaxel | 31.0% | 0.0% | 31.0% |
|  | Gemcitabine/Vinorelbine | 28.0% | 1.9% | 26.1% |
| Osteosarcoma | Cisplatin/Doxorubicin | 41.0% | - | - |
|  | Cisplatin/Doxorubicin | 29.9% | - | - |
|  | Cisplatin/Doxorubicin | 50.0% | - | - |
|  | Methotrexate High Dose/Cisplatin/Doxorubicin | 71.0% | - | - |
|  | Methotrexate High Dose/Cisplatin/Doxorubicin | 60.0% | - | - |
| Soft Tissue Sarcomas | Doxorubicin/Dacarbazine | 17.0% | 5.0% | 12.0% |
|  | Doxorubicin/Dacarbazine | 17.0% | 2.0% | 15.0% |
|  | Doxorubicin/Ifosfamide/Mesna | 34.0% | - | - |
|  | Mesna/Doxorubicin/Ifosfamide/ Dacarbazine | 47.0% | 10.0% | 37.0% |
|  | Gemcitabine/Docetaxel | 52.9% | 8.8% | 44.1% |
|  | Gemcitabine/Docetaxel | 16.0% | - | - |
|  | Gemcitabine/Vinorelbine | 12.5% | 2.5% | 10.0% |
|  | Gemcitabine/Dacarbazine | 12.0% | - | - |
|  | Doxorubicin | 11.9% | - | - |
| Breast Cancer | Doxorubicin | 33.3% | 4.2% | 29.1% |
|  | Doxorubicin | 38.0% | - | - |
|  | Liposomal Doxorubicin | 33.0% | - | - |
|  | Paclitaxel | 32.0% | - | - |
|  | Paclitaxel | 21.5% | 2.3% | 19.2% |
|  | Capecitabine | 38.0% | - | - |
|  | Gemcitabine | 37.1% | - | - |
|  | Vinorelbine | 25.0% | - | - |
|  | Eribuline | 57.0% | 3.0% | 54.0% |
|  | Docetaxel | 34.0% | 2.0% | 32.0% |
|  | Doxorubicin or Epirubicin/Cyclophosphamide or Paclitaxel | 32.0% | - | - |
| Cervical Cancer | Cisplatin/Paclitaxel | 45.0% | - | - |
|  | Cisplatin/Paclitaxel | 29.1% | 2.9% | 26.2% |
|  | Cisplatin/Paclitaxel | 36.0% | 15.0% | 21.0% |
|  | Topotecan/Paclitaxel | 27.0% | - | - |
|  | Carboplatin/Paclitaxel | 53.3% | 26.7% | 26.7% |
|  | Carboplatin/Paclitaxel | 62.6% | 7.1% | 55.5% |
| Uterine Cancer | Carboplatin/Paclitaxel | 75.0% | 25.0% | 50.0% |
| Ovarian Cancer (Epithelial, Fallopian Tube, Peritoneal) | Carboplatin/Gemcitabine | 47.2% | 14.6% | 32.6% |
|  | Carboplatin/Gemcitabine | 57.4% | 9.1% | 48.3% |
|  | Carboplatin/Paclitaxel | 66.0% | - | - |
|  | Carboplatin/Paclitaxel | 59.0% | 18.0% | 41.0% |
| Vulvar Cancer (Squamous Cell Carcinoma) | Cisplatin/Fluorouracil | 60.0% | - | 60.0% |
| Prostate Cancer | Docetaxel | 17.0% | - | 17.0% |
|  | Docetaxel | 12.0% | 12.0% | - |
| Testicular Cancer (Germ Cell Tumors) | Etoposide/Cisplatin | 99.0% | 91.0% | 8.0% |
|  | Bleomycin/Etoposide/Cisplatin | 62.0% | 31.0% | 31.0% |
|  | Etoposide/Ifosfamide/Cisplatin | 60.0% | 37.0% | 22.0% |
| Penile Cancer | Cisplatin/Ifosfamide/Paclitaxel | 50.0% | 10.0% | 40.0% |
| Bladder (Urothelial) Cancer | Gemcitabine/Cisplatin | 49.4% | 12.2% | 37.2% |
|  | Dose-Dense Methotrexate/Vinblastine/Doxorubicin/Cisplatin | 73.0% | 25.0% | 48.0% |
|  | Gemcitabine/Carboplatin | 41.2% | 3.4% | 37.8% |
| Central Nervous System Cancers | Temozolomide | 14.0% | 0.0% | 14.0% |
|  | Lomustine or Carmustine | 4.3% | - | - |
| Hodgkin Lymphoma | Doxorubicin/Bleomycin/Vinblastine/ Dacarbazine | 74.6% | 74.6% | - |
|  | Doxorubicin/Bleomycin/Vinblastine/ Dacarbazine | 73.0% | 73.0% | - |
|  | Doxorubicin/Bleomycin/Vinblastine/ Dacarbazine | 81.7% | 81.7% | - |
|  | Doxorubicin/Vinblastine/ Mechlorethamine/Etoposide/ Vincristine/Bleomycin/Prednisone | 69.0% | 69.0% | - |
| Non-Hodgkin Lymphoma | Cyclophosphamide / Doxorubicin / Vincristine / Prednisone | 68.0% | 68.0% | - |
|  | Cyclophosphamide / Doxorubicin / Vincristine / Prednisone | 44.0% | 44.0% | - |
|  | Cyclophosphamide / Doxorubicin / Vincristine / Prednisone | 63.0% | 63.0% | - |
|  | Cyclophosphamide / Doxorubicin / Vincristine / Prednisone | 76.0% | 76.0% | - |
|  | Cyclophosphamide / Doxorubicin / Vincristine / Prednisone | 82.2% | 82.2% | - |
|  | Cyclophosphamide / Doxorubicin / Vincristine / Prednisone | 76.1% | 76.1% | - |
|  | Cyclophosphamide / Doxorubicin / Vincristine / Prednisone | 68.0% | 68.0% | - |
|  | Cyclophosphamide / Doxorubicin / Vincristine / Prednisone | 62.3% | 62.3% | - |
|  | Cyclophosphamide / Doxorubicin / Vincristine / Intrathecal Methotrexate and Cytarabine / High Dose Systemic Methotrexate | 86.0% | 86.0% | - |
|  | Cyclophosphamide / Doxorubicin / Vincristine / Intrathecal Methotrexate and Cytarabine / High Dose Systemic Methotrexate | 85.0% | 85.0% | - |
|  | Cyclophosphamide / Doxorubicin / Vincristine / Intrathecal Methotrexate and Cytarabine / High Dose Systemic Methotrexate | 92.0% | 92.0% | - |
|  | Cyclophosphamide / Doxorubicin / Etoposide / Vincristine / Prednisone | 90.4% | 90.4% | - |
|  | Cyclophosphamide / Doxorubicin / Etoposide / Vincristine / Prednisone | 71.6% | 71.6% | - |
| Acute Lymphoblastic Leukemia | Daunorubicin / Vincristine / Prednisone / Pegaspargase / Cyclophosphamide | 93.5% | 93.5% | - |
|  | Daunorubicin / Vincristine / Prednisone / Pegaspargase | 93.0% | 93.0% | - |
|  | Daunorubicin / Vincristine / Prednisone / Pegaspargase / Cyclophosphamide | 85.0% | 85.0% | - |
|  | Daunorubicin / Vincristine / Prednisone / Pegaspargase (induction phase I); and Cyclophosphamide/ Cytarabine | 91.0% | 91.0% | - |
| Chronic Lymphocytic Leukemia / Small Lymphocytic Leukemia | Fludarabine / Cyclophosphamide | 58.0% | 13.0% | 44.9% |
| Acute Myeloid Leukemia | Daunorubicin / Cytarabine | 70.6% | 70.6% | - |
|  | Daunorubicin / Cytarabine | 71.0% | 71.0% | - |
|  | Daunorubicin (60 mg/m2)/ Cytarabine | 84.0% | 84.0% | - |
|  | Daunorubicin (90 mg/m2)/ Cytarabine | 81.0% | 81.0% | - |
|  | Daunorubicin or Idarubicin | 77.0% | 77.0% | - |

^a.^ Refer to references 1-135 for corresponding primary clinical trial from which the response rates were obtained.

**Supplemental Table 2. List of Cancer Types Deemed Ineligible to Achieve Response from Cytotoxic Chemotherapy**.

| **Cancer Site** | **Rationale for Exclusion** |
| --- | --- |
| "Other" Digestive Organs | Mortality data did not specify what types of cancers were included in this category. |
| "Other" Female Genital | Mortality data did not specify what types of cancers were included in this category |
| "Other" Respiratory Organs | Mortality data did not specify what types of cancers were included in this category. |
| Adult Intracranial and Spinal Ependymoma (excluding Subependymoma) | Mainstay of therapy includes surgery. |
| Adult Low-Grade Infiltrative Supratentorial Astrocytoma/Oligodendroglioma | Mainstay of therapy includes surgery; systemic chemotherapy often in adjuvant setting. |
| Adult Medulloblastoma | Mainstay of therapy includes surgery; systemic chemotherapy often concurrently with radiation. |
| AIDS-Related Kaposi Sarcoma | Uncommon malignancy. |
| Alveolar Soft Part Sarcoma | Uncommon malignancy; mainstay of therapy is sunitinib. |
| Anaplastic Glioma | Mainstay of therapy includes surgery; systemic chemotherapy often in adjuvant setting. |
| Angiosarcoma | Uncommon malignancy; mainstay of therapy includes platinum-based therapies, vinorelbine, sorafenib, sunitinib, or bevacizumab. |
| Cancers of the Eye/Orbit (Uveal Melanoma) | Mainstay of therapy includes immunotherapy or targeted therapy (although clinical trial preferred). |
| Chondrosarcoma | Chemotherapy regimens were not NCCN "preferred" regimens (i.e. category 1 or 2A). |
| Chordoma | Mainstay of therapy includes imatinib, erlotinib, sunitinib, lapatinib, sorafenib, or dasatinib. |
| Chronic Myeloid Leukemia | Preferred regimen includes tyrosine kinase inhibitors. |
| Cutaneous Basal Cell or Squamous Cell Carcinoma | No corresponding mortality data for analysis. |
| Cutaneous Melanoma | Mainstay of therapy includes anti-PD1 monotherapy (i.e., pembrolizumab) or targeted therapy (BRAF V600 mutation) |
| Dermatofibrosarcoma Protuberans | Mainstay of therapy includes surgical excision. |
| Desmoid Tumors (Aggressive Fibromatosis) | Uncommon malignancy; mainly endocrinologic agents, tyrosine kinase inhibitors, or other chemotherapy. |
| Ewing Sarcoma | Mainly affecting children, thus unlikely to cause significant mortality. |
| Extranodal NK/T-Cell Lymphoma, Nasal Type | Uncommon malignancy. |
| Follicular Lymphoma | Preferred regimen includes rituximab or obinutuzumab. |
| Gastrointestinal Stromal Tumors (GIST) | Mainstay of treatment is imatinib, which is not a cytotoxic chemotherapy agent |
| Gestational Trophoblastic Neoplasia | Uncommon malignancy. |
| Giant Cell Tumor of Bone | Mainstay of therapy includes denosumab or interferon alfa-2b. |
| Hairy Cell Leukemia | Uncommon malignancy. |
| Hepatocellular Carcinoma | Mainstay of therapy often includes surgery and/or radiation/ablative procedures. |
| Inflammatory Myofibroblastic Tumor with ALK Translocation | Uncommon malignancy; mainstay of therapy includes crizotinib, ceritinib. |
| Kidney and Renal Pelvis (Clear Cell and Non-Clear Cell Carcinoma) | Mainstay of therapy includes surgery. |
| Lymphangioleiomyomatosis | Uncommon malignancy; mainstay of therapy includes sirolimus, everolimus, temsirolimus. |
| Mantle Cell Lymphoma | Preferred regimens include rituximab. |
| Marginal Zone Lymphoma | Preferred regimens include rituximab. |
| Meningioma | Mainstay of therapy includes surgery and radiation therapy; no "preferred" systemic therapies. |
| Merkel Cell Carcinoma | Uncommon malignancy; mainstay of therapy includes avelumab, pembrolizumab, or nivolumab. |
| Multiple Myeloma | Mainstay of therapy includes bortezomib. |
| Mycosis Fungoides / Sezary Syndrome | Uncommon malignancy. |
| Myelodysplastic Syndromes | Mainstay of therapy includes immunosuppressive therapy, but have chemotherapy (azacytidine, decitabine, or lenalidomide) if no good response. |
| Myeloproliferative Syndromes | Mainstay of therapy includes ruxolitinib or clinical trial. |
| Neuroendocrine/Adrenal Neoplasms | Preferred regimens include somatostatin analogues or peptide receptor radionuclide therapy (PRRT). |
| Non-Pleomorphic Rhabdomyosarcoma | Uncommon malignancy. |
| Perivascular Epitheloid Cell Neoplasm (PEComa) | Uncommon malignancy; mainstay of therapy includes sirolimus, everolimus, temsirolimus. |
| Ph-Positive Acute Lymphoblastic Leukemia | Preferred regimen includes tyrosine kinase inhibitors. |
| Pigmented Villonodular Synovitis/Tenosynovial Giant Cell Tumor | Uncommon Malignancy; mainstay of therapy is imatinib. |
| Primary CNS Lymphoma | Preferred regimen includes rituximab or radiation therapy. |
| Recurrent Angiomyolipoma | Uncommon malignancy; mainstay of therapy includes sirolimus, everolimus, temsirolimus. |
| Small Intestine | No corresponding NCCN Guideline for small intestinal malignancies. |
| Solitary Fibrous Tumor/Hemangiopericytoma | Uncommon malignancy; mainstay of therapy is bevacizumab + temozolomide, sunitinib, or sorafenib. |
| Systemic Light Chain Amyloidosis | Mainstay of therapy includes bortezomib. |
| Systemic Mastocytosis | Uncommon malignancy; but advanced disease can undergo clinical trial, midostaurin, cladribine, imatinib, or interferons. |
| Thymoma and Thymic Malignancies | Uncommon malignancy. |
| Thyroid Cancer (Papillary, Follicular, Hurthle Cell, Medullary, Anaplastic) | Mainstay of therapy includes surgery, radioactive iodine ablation, endocrinologic agents, or specific antibodies. |
| Ureter and "Other" Urinary Organs | No corresponding NCCN guideline for ureter malignancies; Mortality data did not specify what types of cancers were included in this category. |
| Uterine (Endometrial) Cancer | Mainstay of therapy includes surgery, with only adjuvant chemotherapy preferred. |
| Vagina | No corresponding NCCN Guideline for vaginal malignancies. |
| Waldenstrom Macroglobulinemia / Lymphoplasmacytic Lymphoma | Mainstay of therapy includes rituximab, bortezomib. |

**References**

1. Vermorken JB, Mesia R, Rivera F, et al. Platinum-based chemotherapy plus cetuximab in head and neck cancer. N Engl J Med. 2008;359(11):1116–1127.

2. Hui EP, Ma BB, Leung SF. et al. Randomized phase II trial of concurrent cisplatin-radiotherapy with or without neoadjuvant docetaxel and cisplatin in advanced nasopharyngeal carcinoma. J Clin Oncol. 2009;27:242–249.

3. Samlowski WE, Moon J, Kuebler JP, et al. Evaluation of the combination of docetaxel/carboplatin in patients with metastatic or recurrent squamous cell carcinoma of the head and neck (SCCHN): a Southwest Oncology Group Phase II study. Cancer Invest. 2007;Apr-May;25(3):182-8.

4. Gibson MK, Li Y, Murphy B, et al. Randomized phase III evaluation of cisplatin plus fluorouracil versus cisplatin plus paclitaxel in advanced head and neck cancer (E1395): an intergroup trial of the Eastern Cooperative Oncology Group. J Clin Oncol. 2005;23(15):3562-7.

5. Jin Y, Shi YX, Cai XY, et al. Comparison of five cisplatin-based regimens frequently used as the first-line protocols in metastatic nasopharyngeal carcinoma. J Cancer Res Clin Oncol. 2012;138(10):1717-25. Epub 2012 Jun 10. Erratum in: J Cancer Res Clin Oncol. 2015;141(4):767.

6. Burtness B, Goldwasser MA, Flood W, Mattar B, Forastiere AA; Eastern Cooperative Oncology Group. Phase III randomized trial of cisplatin plus placebo compared with cisplatin plus cetuximab in metastatic/recurrent head and neck cancer: an Eastern Cooperative Oncology Group study. J Clin Oncol. 2005;23(34):8646-54. Erratum in: J Clin Oncol. 2006;24(4):724.

7. Gibson MK, Li Y, Murphy B, et al. Randomized phase III evaluation of cisplatin plus fluorouracil versus cisplatin plus paclitaxel in advanced head and neck cancer (E1395): an intergroup trial of the Eastern Cooperative Oncology Group. J Clin Oncol. 2005;23(15):3562-7.

8. Forastiere AA, Metch B, Schuller DE, et al. Randomized comparison of cisplatin plus fluorouracil and carboplatin plus fluorouracil versus methotrexate in advanced squamous-cell carcinoma of the head and neck: a Southwest Oncology Group study. J Clin Oncol. 1992;10(8):1245-51.

9. Jin Y, Shi YX, Cai XY, et al .Comparison of five cisplatin-based regimens frequently used as the first-line protocols in metastatic nasopharyngeal carcinoma. J Cancer Res Clin Oncol. 2012;138(10):1717-25. Epub 2012 Jun 10. Erratum in: J Cancer Res Clin Oncol. 2015;141(4):767.

10. Zhang L, Huang Y, Hong S, et al. Gemcitabine plus cisplatin versus fluorouracil plus cisplatin in recurrent or metastatic nasopharyngeal carcinoma: a multicentre, randomised, open-label, phase 3 trial. Lancet. 2016 Oct15;388(10054):1883-1892. Erratum in: Lancet. 2016 Oct 15;388(10054):1882.

11. Jin Y, Shi YX, Cai XY, et al. Comparison of five cisplatin-based regimens frequently used as the first-line protocols in metastatic nasopharyngeal carcinoma. J Cancer Res Clin Oncol. 2012;138(10):1717-25. Erratum in: J Cancer Res Clin Oncol. 2015;141(4):767.

12. Zhang L, Huang Y, Hong S, et al. Gemcitabine plus cisplatin versus fluorouracil plus cisplatin in recurrent or metastatic nasopharyngeal carcinoma: a multicentre, randomised, open-label, phase 3 trial. Lancet. 2016;388(10054):1883-1892. Erratum in: Lancet. 2016;388(10054):1882.

13. Bang YJ, Van Cutsem E, Feyereislova A, et al. Trastuzumab in combination with chemotherapy versus chemotherapy alone for treatment of HER2-positive advanced gastric orgastro-oesophageal junction cancer (ToGA): a phase 3, open-label, randomized controlled trial. Lancet. 2010;376(9742):687-97. Erratum in: Lancet. 2010;376(9749):1302.

14. Lorenzen S, Schuster T, Porschen R, et al. Cetuximab plus cisplatin-5-fluorouracil versus cisplatin-5-fluorouracil alone in first-line metastatic squamous cell carcinoma of the esophagus: a randomized phase II study of the ArbeitsgemeinschaftInternistische Onkologie. Ann Oncol. 2009;20(10):1667-73.

15. Al-Batran SE, Hartmann JT, Probst S, et al. Phase III trial in metastatic gastroesophageal adenocarcinoma with fluorouracil, leucovorin plus either oxaliplatin or cisplatin: a study of the Arbeitsgemeinschaft Internistische Onkologie. J Clin Oncol. 2008;26(9):1435-42.

16. Bouché O, Raoul JL, Bonnetain F, et al. Randomized multicenter phase II trial of a biweekly regimen of fluorouracil and leucovorin (LV5FU2), LV5FU2 plus cisplatin, or LV5FU2 plus irinotecan in patients with previously untreated metastatic gastric cancer: a Federation Francophone de Cancerologie Digestive Group Study--FFCD 9803. J Clin Oncol. 2004;22(21):4319-28.

17. Kang YK, Kang WK, Shin DB, et al. Capecitabine/cisplatin versus 5-fluorouracil/cisplatin as first-line therapy in patients with advanced gastric cancer: a randomised phase III noninferiority trial. Ann Oncol. 2009;20(4):666-73.

18. Al-Batran SE, Hartmann JT, Probst S, et al. Phase III trial in metastatic gastroesophageal adenocarcinoma with fluorouracil, leucovorin plus either oxaliplatin or cisplatin: a study of the Arbeitsgemeinschaft Internistische Onkologie. J Clin Oncol. 2008;26(9):1435-42.

19. Kim GM, Jeung HC, Rha SY, et al. A randomized phase II trial of S-1-oxaliplatin versus capecitabine-oxaliplatin in advanced gastric cancer. Eur J Cancer. 2012;48(4):518-26.

20. Bang YJ, Van Cutsem E, Feyereislova A, et al. Trastuzumab in combination with chemotherapy versus chemotherapy alone for treatment of HER2-positive advanced gastric orgastro-oesophageal junction cancer (ToGA): a phase 3, open-label, randomised controlled trial. Lancet. 2010;376(9742):687-97. Erratum in: Lancet. 2010;376(9749):1302.

21. Lorenzen S, Schuster T, Porschen R, et al. Cetuximab plus cisplatin-5-fluorouracil versus cisplatin-5-fluorouracil alone in first-line metastatic squamous cell carcinoma of the esophagus: a randomized phase II study of the ArbeitsgemeinschaftInternistische Onkologie. Ann Oncol. 2009;20(10):1667-73.

22. Al-Batran SE, Hartmann JT, Probst S, et al. Phase III trial in metastatic gastroesophageal adenocarcinoma with fluorouracil, leucovorin plus either oxaliplatin or cisplatin: a study of the Arbeitsgemeinschaft Internistische Onkologie. J Clin Oncol. 2008;26(9):1435-42.

23. Bouché O, Raoul JL, Bonnetain F, et al. Randomized multicenter phase II trial of a biweekly regimen of fluorouracil and leucovorin (LV5FU2), LV5FU2 plus cisplatin, or LV5FU2 plus irinotecan in patients with previously untreated metastatic gastric cancer: a Federation Francophone de Cancerologie Digestive Group Study--FFCD 9803. J Clin Oncol. 2004;22(21):4319-28.

24. Kang YK, Kang WK, Shin DB, et al. Capecitabine/cisplatin versus 5-fluorouracil/cisplatin as first-line therapy in patients with advanced gastric cancer: a randomised phase III noninferiority trial. Ann Oncol. 2009;20(4):666-73.

25. Al-Batran SE, Hartmann JT, Probst S, et al. Phase III trial in metastatic gastroesophageal adenocarcinoma with fluorouracil, leucovorin plus either oxaliplatin or cisplatin: a study of the Arbeitsgemeinschaft Internistische Onkologie. J Clin Oncol. 2008;26(9):1435-42.

26. Kim GM, Jeung HC, Rha SY, et al. A randomized phase II trial of S-1-oxaliplatin versus capecitabine-oxaliplatin in advanced gastric cancer. Eur J Cancer. 2012;48(4):518-26.

27. de Gramont A, Figer A, Seymour M, et al. Leucovorin and fluorouracil with or without oxaliplatin as first-line treatment in advanced colorectal cancer. J Clin Oncol. 2000;18(16):2938-47.

28. Cheeseman SL, Joel SP, Chester JD, et al. A 'modified de Gramont' regimen of fluorouracil, alone and with oxaliplatin, for advanced colorectal cancer. Br J Cancer. 2002;87(4):393-9.

29. Maindrault-Goebel F, de Gramont A, Louvet C, et al. Evaluation of oxaliplatin dose intensity in bimonthly leucovorin and 48-hour 5-fluorouracil continuous infusion regimens (FOLFOX) in pretreated metastatic colorectal cancer. Oncology Multidisciplinary Research Group (GERCOR). Ann Oncol. 2000;11(11):1477-83.

30. Douillard JY, Siena S, Cassidy J, et al. Randomized, phase III trial of panitumumab with infusional fluorouracil, leucovorin, and oxaliplatin (FOLFOX4) versus FOLFOX4 alone as first-line treatment in patients with previously untreated metastatic colorectal cancer: the PRIME study. J Clin Oncol. 2010;28(31):4697-705.

31. Saltz LB, Clarke S, Díaz-Rubio E, et al. Bevacizumab in combination with oxaliplatin-based chemotherapy as first-line therapy in metastatic colorectal cancer: a randomized phase III study. J Clin Oncol. 2008;26(12):2013-9.

32. Fuchs CS, Marshall J, Mitchell E, et al. Randomized, controlled trial of irinotecan plus infusional, bolus, or oral fluoropyrimidines in first-line treatment of metastatic colorectal cancer: results from the BICC-C Study. J Clin Oncol. 2007;25(30):4779-86.

33. Falcone A, Ricci S, Brunetti I, et al. Phase III trial of infusional fluorouracil, leucovorin, oxaliplatin, and irinotecan (FOLFOXIRI) compared with infusional fluorouracil, leucovorin, and irinotecan (FOLFIRI) as first-line treatment for metastatic colorectal cancer: the Gruppo Oncologico Nord Ovest. J Clin Oncol. 2007;25(13):1670-6.

34. Cunningham D, Lang I, Marcuello E, et al. Bevacizumab plus capecitabine versus capecitabine alone in elderly patients with previously untreated metastatic colorectal cancer (AVEX): an open-label, randomised phase 3 trial. Lancet Oncol. 2013;14(11):1077-1085.

35. Eng C, Chang GJ, You YN, et al. The role of systemic chemotherapy and multidisciplinary management in improving the overall survival of patients with metastatic squamous cell carcinoma of the anal canal. Oncotarget. 2014;5(22):11133-42.

36. Faivre C, Rougier P, Ducreux M, et al. 5-fluorouracile and cisplatinum combination chemotherapy for metastatic squamous-cell anal cancer. Bull Cancer. 1999 ;86(10):861-5.

37. Eng C, Chang GJ, You YN, et al. The role of systemic chemotherapy and multidisciplinary management in improving the overall survival of patients with metastatic squamous cell carcinoma of the anal canal. Oncotarget. 2014;5(22):11133-42.

38. Valle J, Wasan H, Palmer DH, et al. Cisplatin plus gemcitabine versus gemcitabine for biliary tract cancer. N Engl J Med. 2010;362(14):1273-81.

39. Conroy T, Desseigne F, Ychou M, et al. FOLFIRINOX versus gemcitabine for metastatic pancreatic cancer. N Engl J Med. 2011;364(19):1817-25.

40. Von Hoff DD, Ervin T, Arena FP, et al. Increased survival in pancreatic cancer with nab-paclitaxel plus gemcitabine. N Engl J Med. 2013;369(18):1691-703.

41. Moore MJ, Goldstein D, Hamm J, et al. Erlotinib plus gemcitabine compared with gemcitabine alone in patients with advanced pancreatic cancer: a phase III trial of the National Cancer Institute of Canada Clinical Trials Group. J Clin Oncol. 2007;25(15):1960-6.

42. Cunningham D, Chau I, Stocken DD, et al. Phase III randomized comparison of gemcitabine versus gemcitabine plus capecitabine in patients with advanced pancreatic cancer. J Clin Oncol. 2009;27(33):5513-8.

43. Horn L, Mansfield AS, Szczęsna A, et al. First-Line Atezolizumab plus Chemotherapy in Extensive-Stage Small-Cell Lung Cancer. N Engl J Med. 2018;379(23):2220-2229.

44. Okamoto H, Watanabe K, Nishiwaki Y, et al. Phase II study of area under the plasma-concentration-versus-time curve-based carboplatin plus standard-dose intravenous etoposide in elderly patients with small-cell lung cancer. J Clin Oncol. 1999;17(11):3540-5.

45. Spigel DR, Townley PM, Waterhouse DM, et al. Randomized phase II study of bevacizumab in combination with chemotherapy in previously untreated extensive-stage small-cell lung cancer: results from the SALUTE trial. J Clin Oncol. 2011;29(16):2215-22.

46. Evans WK, Shepherd FA, Feld R, Osoba D, DeBoer G. First-line therapy withVP-16 and cisplatin for small-cell lung cancer. Semin Oncol. 1986;13(3 Suppl 3):17-23.

47. Niell HB, Herndon JE, Miller AA, et al. Randomized phase III intergroup trial of etoposide and cisplatin with or without paclitaxel and granulocyte colony-stimulating factor in patients with extensive-stage small-cell lung cancer: Cancer and Leukemia Group B Trial 9732. J Clin Oncol. 2005;23(16):3752-9.

48. Schmittel A, Fischer von Weikersthal L, Sebastian M, et al. A randomized phase II trial of irinotecan plus carboplatin versus etoposide plus carboplatin treatment in patients with extended disease small-cell lung cancer. Ann Oncol. 2006;17(4):663-7.

49. Noda K, Nishiwaki Y, Kawahara M, et al. Irinotecan plus cisplatin compared with etoposide plus cisplatin for extensive small-cell lung cancer. N Engl J Med. 2002;346(2):85-91.

50. Hanna N, Bunn PA Jr, Langer C, et al. Randomized phase III trial comparing irinotecan/cisplatin with etoposide/cisplatin in patients with previously untreated extensive-stage disease small-cell lung cancer. J Clin Oncol. 2006;24(13):2038-43.

51. Socinski MA, Bondarenko I, Karaseva NA, et al. Weekly nab-paclitaxel in combination with carboplatin versus solvent-based paclitaxel plus carboplatin as first-line therapy in patients with advanced non-small-cell lung cancer: final results of a phase III trial. J Clin Oncol. 2012;30(17):2055-62.

52. Paz-Ares L, Luft A, Vicente D, et al. Pembrolizumab plus Chemotherapy for Squamous Non-Small-Cell Lung Cancer. N Engl J Med. 2018;379(21):2040-2051.

53. Fossella F, Pereira JR, von Pawel J, et al. Randomized, multinational, phase III study of docetaxel plus platinum combinations versus vinorelbine plus cisplatin for advanced non-small-cell lung cancer: the TAX 326 study group. J Clin Oncol. 2003;21(16):3016-24.

54. Klastersky J, Sculier JP, Lacroix H, et al. A randomized study comparing cisplatin or carboplatin with etoposide in patients with advanced non-small-cell lung cancer: European Organization for Research and Treatment of Cancer Protocol 07861. J Clin Oncol. 1990;8(9):1556-62.

55. Frasci G, Comella P, Panza N, et al. Carboplatin-oral etoposide personalized dosing in elderly non-small cell lung cancer patients. Gruppo Oncologico Cooperativo Sud-Italia. Eur J Cancer. 1998;34(11):1710-4.

56. Danson S, Middleton MR, O'Byrne KJ, et al. Phase III trial of gemcitabine and carboplatin versus mitomycin, ifosfamide, and cisplatin or mitomycin, vinblastine, and cisplatin in patients with advanced nonsmall cell lung carcinoma. Cancer. 2003;98(3):542-53.

57. Ohe Y, Ohashi Y, Kubota K, et al. Randomized phase III study of cisplatin plus irinotecan versus carboplatin plus paclitaxel, cisplatin plus gemcitabine, and cisplatin plus vinorelbine for advanced non-small-cell lung cancer: Four-Arm Cooperative Study in Japan. Ann Oncol. 2007;18(2):317-23.

58. Scagliotti GV, Kortsik C, Dark GG, et al. Pemetrexed combined with oxaliplatin or carboplatin as first-line treatment in advanced non-small cell lung cancer: a multicenter, randomized, phase II trial. Clin Cancer Res. 2005;11(2 Pt 1):690-6.

59. Langer CJ, Gadgeel SM, Borghaei H, et al. Carboplatin and pemetrexed with or without pembrolizumab for advanced, non-squamous non-small-cell lung cancer: a randomised, phase 2 cohort of the open-label KEYNOTE-021 study. Lancet Oncol. 2016;17(11):1497-1508.

60. Gandhi L, Rodríguez-Abreu D, Gadgeel S, et al. Pembrolizumab plus Chemotherapy in Metastatic Non-Small-Cell Lung Cancer. N Engl J Med. 2018;378(22):2078-2092.

61. Cardenal F, López-Cabrerizo MP, Antón A, et al. Randomized phase III study of gemcitabine-cisplatin versus etoposide-cisplatin in the treatment of locally advanced or metastatic non-small-cell lung cancer. J Clin Oncol. 1999;17(1):12-8.

62. Scagliotti GV, Parikh P, von Pawel J, et al. Phase III study comparing cisplatin plus gemcitabine with cisplatin plus pemetrexed in chemotherapy-naive patients with advanced-stage non-small-cell lung cancer. J Clin Oncol. 2008;26(21):3543-51.

63. Schiller JH, Harrington D, Belani CP, et al. Comparison of four chemotherapy regimens for advanced non-small-cell lung cancer. N Engl J Med. 2002;346(2):92-8.

64. Pujol JL, Breton JL, Gervais R, et al. Gemcitabine-docetaxel versus cisplatin-vinorelbine in advanced or metastatic non-small-cell lung cancer: a phase III study addressing the case for cisplatin. Ann Oncol. 2005;16(4):602-10.

65. Tan EH, Szczesna A, Krzakowski M, et al. Randomized study of vinorelbine-gemcitabine versus vinorelbine-carboplatin in patients with advanced non-small cell lung cancer. Lung Cancer. 2005;49(2):233-40.

66. Bramwell VH, Burgers M, Sneath R, et al. A comparison of two short intensive adjuvant chemotherapy regimens in operable osteosarcoma of limbs in children and young adults: the first study of the European Osteosarcoma Intergroup. J Clin Oncol. 1992;10(10):1579-91.

67. Souhami RL, Craft AW, Van der Eijken JW, Nooij M, et al. Randomised trial of two regimens of chemotherapy in operable osteosarcoma: a study of the European Osteosarcoma Intergroup. Lancet. 1997;350(9082):911-7.

68. Lewis IJ, Nooij MA, Whelan J, et al. Improvement in histologic response but not survival in osteosarcoma patients treated with intensified chemotherapy: a randomized phase III trial of the European Osteosarcoma Intergroup. J Natl Cancer Inst. 2007;99(2):112-28.

69. Bacci G, Ferrari S, Bertoni F, et al. Long-term outcome for patients with nonmetastatic osteosarcoma of the extremity treated at the istituto ortopedico rizzoli according to the istituto ortopedico rizzoli/osteosarcoma-2 protocol: an updated report. J Clin Oncol. 2000;18(24):4016-27.

70. Winkler K, Beron G, Delling G, et al. Neoadjuvant chemotherapy of osteosarcoma: results of a randomized cooperative trial (COSS-82) with salvage chemotherapy based on histological tumor response. J Clin Oncol. 1988;6(2):329-37.

71. Zalupski M, Metch B, Balcerzak S, et al. Phase III comparison of doxorubicin and dacarbazine given by bolus versus infusion in patients with soft-tissue sarcomas: a Southwest Oncology Group study. J Natl Cancer Inst. 1991;83(13):926-32.

72. Antman K, Crowley J, Balcerzak SP, et al. An intergroup phase III randomized study of doxorubicin and dacarbazine with or without ifosfamide and mesna in advanced soft tissue and bone sarcomas. J Clin Oncol. 1993;11(7):1276-85.

73. Edmonson JH, Ryan LM, Blum RH, et al. Randomized comparison of doxorubicin alone versus ifosfamide plus doxorubicin or mitomycin, doxorubicin, and cisplatin against advanced soft tissue sarcomas. J Clin Oncol. 1993;11(7):1269-75.

74. Elias A, Ryan L, Sulkes A, Collins J, Aisner J, Antman KH. Response to mesna, doxorubicin, ifosfamide, and dacarbazine in 108 patients with metastatic or unresectable sarcoma and no prior chemotherapy. J Clin Oncol. 1989;7(9):1208-16.

75. Hensley ML, Maki R, Venkatraman E, et al. Gemcitabine and docetaxel in patients with unresectable leiomyosarcoma: results of a phase II trial. J Clin Oncol. 2002;20(12):2824-31.

76. Maki RG, Wathen JK, Patel SR, et al. Randomized phase II study of gemcitabine and docetaxel compared with gemcitabine alone in patients with metastatic soft tissue sarcomas: results of sarcoma alliance for research through collaboration study 002 [corrected]. J Clin Oncol. 2007;25(19):2755-63. Erratum in: J Clin Oncol. 2007;25(24):3790.

77. Dileo P, Morgan JA, Zahrieh D, et al. Gemcitabine and vinorelbine combination chemotherapy for patients with advanced soft tissue sarcomas: results of a phase II trial. Cancer. 2007;109(9):1863-9.

78. García-Del-Muro X, López-Pousa A, Maurel J, et al. Randomized phase II study comparing gemcitabine plus dacarbazine versus dacarbazine alone in patients with previously treated soft tissue sarcoma: a Spanish Group for Research on Sarcomas study. J Clin Oncol. 2011;29(18):2528-33.

79. Tap WD, Jones RL, Van Tine BA, et al. Olaratumab and doxorubicin versus doxorubicin alone for treatment of soft-tissue sarcoma: an open-label phase 1b and randomised phase 2 trial. Lancet. 2016;388(10043):488-97. Erratum in: Lancet. 2016;388(10043):464.

80. Chan S, Friedrichs K, Noel D, et al. Prospective randomized trial of docetaxel versus doxorubicin in patients with metastatic breast cancer. J Clin Oncol. 1999;17(8):2341-54.

81. Gasparini G, Dal Fior S, Panizzoni GA, Favretto S, Pozza F. Weekly epirubicin versus doxorubicin as second line therapy in advanced breast cancer. A randomized clinical trial. Am J Clin Oncol. 1991;14(1):38-44.

82. O'Brien ME, Wigler N, Inbar M, et al. Reduced cardiotoxicity and comparable efficacy in a phase III trial of pegylated liposomal doxorubicin HCl (CAELYX/Doxil) versus conventional doxorubicin for first-line treatment of metastatic breast cancer. Ann Oncol. 2004;15(3):440-9.

83. Seidman AD, Tiersten A, Hudis C, et al. Phase II trial of paclitaxel by 3-hourinfusion as initial and salvage chemotherapy for metastatic breast cancer. J Clin Oncol. 1995;13(10):2575-81.

84. Perez EA, Vogel CL, Irwin DH, Kirshner JJ, Patel R. Multicenter phase II trial of weekly paclitaxel in women with metastatic breast cancer. J Clin Oncol. 2001;19(22):4216-23.

85. Bajetta E, Procopio G, Celio L, et al. Safety and efficacy of two different doses of capecitabine in the treatment of advanced breast cancer in older women. J Clin Oncol. 2005;23(10):2155-61.

86. Seidman AD. Gemcitabine as single-agent therapy in the management of advanced breast cancer. Oncology (Williston Park). 2001;15(2 Suppl 3):11-4.

87. Zelek L, Barthier S, Riofrio M, et al. Weekly vinorelbine is an effective palliative regimen after failure with anthracyclines and taxanes in metastatic breast carcinoma. Cancer. 2001;92(9):2267-72.

88. Cortes J, O'Shaughnessy J, Loesch D, et al. Eribulin monotherapy versus treatment of physician's choice in patients with metastatic breast cancer (EMBRACE): a phase 3 open-label randomized study. Lancet. 2011;377(9769):914-23.

89. Marty M, Cognetti F, Maraninchi D, et al. Randomized phase II trial of the efficacy and safety of trastuzumab combined with docetaxel in patients with human epidermal growth factor receptor 2-positive metastatic breast cancer administered as first-line treatment: the M77001 study group. J Clin Oncol. 2005;23(19):4265-74.

90. Slamon DJ, Leyland-Jones B, Shak S, et al. Use of chemotherapy plus a monoclonal antibody against HER2 for metastatic breast cancer that overexpresses HER2. N Engl J Med. 2001;344(11):783-92.

91. Tewari KS, Sill MW, Long HJ, et al. Improved survival with bevacizumab in advanced cervical cancer. N Engl J Med. 2014 ;370(8):734-43. Erratum in: N Engl J Med. 2017;377(7):702.

92. Monk BJ, Sill MW, McMeekin DS, et al. Phase III trial of four cisplatin-containing doublet combinations in stage IVB, recurrent, or persistent cervical carcinoma: a Gynecologic Oncology Group study. J Clin Oncol. 2009;27(28):4649-55.

93. Moore DH, Blessing JA, McQuellon RP, et al. Phase III study of cisplatin with or without paclitaxel in stage IVB, recurrent, or persistent squamous cell carcinoma of the cervix: a gynecologic oncology group study. J Clin Oncol. 2004;22(15):3113-9.

94. Moore KN, Herzog TJ, Lewin S, et al. A comparison of cisplatin/paclitaxel and carboplatin/paclitaxel in stage IVB, recurrent or persistent cervical cancer. Gynecol Oncol. 2007;105(2):299-303.

95. Kitagawa R, Katsumata N, Shibata T, et al. Paclitaxel Plus Carboplatin Versus Paclitaxel Plus Cisplatin in Metastatic or Recurrent Cervical Cancer: The Open-Label Randomized Phase III Trial JCOG0505. J Clin Oncol. 2015;33(19):2129-35.

96. Fader AN, Roque DM, Siegel E, et al. Randomized Phase II Trial of Carboplatin-Paclitaxel Versus Carboplatin-Paclitaxel-Trastuzumab in Uterine Serous Carcinomas That Overexpress Human Epidermal Growth Factor Receptor 2/neu. J Clin Oncol. 2018;36(20):2044-2051.

97. Pfisterer J, Plante M, Vergote I, et al. Gemcitabine plus carboplatin compared with carboplatin in patients with platinum-sensitive recurrent ovarian cancer: an intergroup trial of the AGO-OVAR, the NCIC CTG, and the EORTC GCG. J Clin Oncol. 2006;24(29):4699-707.

98. Aghajanian C, Blank SV, Goff BA, et al. OCEANS: a randomized, double-blind, placebo-controlled phase III trial of chemotherapy with or without bevacizumab in patients with platinum-sensitive recurrent epithelial ovarian, primary peritoneal, or fallopian tube cancer. J Clin Oncol. 2012;30(17):2039-45.

99. Parmar MK, Ledermann JA, Colombo N, et al. Paclitaxel plus platinum-based chemotherapy versus conventional platinum-based chemotherapy in women with relapsed ovarian cancer: the ICON4/AGO-OVAR-2.2 trial. Lancet. 2003;361(9375):2099-106.

100. Coleman RL, Brady MF, Herzog TJ, et al. Bevacizumab and paclitaxel-carboplatin chemotherapy and secondary cytoreduction in recurrent, platinum-sensitive ovarian cancer (NRG Oncology/Gynecologic Oncology Group study GOG-0213): a multicentre, open-label, randomised, phase 3 trial. Lancet Oncol. 2017;18(6):779-791.

101. Reade CJ, Eiriksson LR, Mackay H. Systemic therapy in squamous cell carcinoma of the vulva: current status and future directions. Gynecol Oncol. 2014;132(3):780-9.

102. Petrylak DP, Tangen CM, Hussain MH, et al. Docetaxel and estramustine compared with mitoxantrone and prednisone for advanced refractory prostate cancer. N Engl J Med. 2004;351(15):1513-20.

103. Tannock IF, de Wit R, Berry WR, et al. Docetaxel plus prednisone or mitoxantrone plus prednisone for advanced prostate cancer. N Engl J Med. 2004;351(15):1502-12.

104. Xiao H, Mazumdar M, Bajorin DF, et al. Long-term follow-up of patients with good-risk germ cell tumors treated with etoposide and cisplatin. J Clin Oncol. 1997;15(7):2553-8.

105. Nichols CR, Catalano PJ, Crawford ED, Vogelzang NJ, Einhorn LH, Loehrer PJ. Randomized comparison of cisplatin and etoposide and either bleomycin or ifosfamide in treatment of advanced disseminated germ cell tumors: an Eastern Cooperative Oncology Group, Southwest Oncology Group, and Cancer and Leukemia Group B Study. J Clin Oncol. 1998;16(4):1287-93.

106. Pagliaro LC, Williams DL, Daliani D, et al. Neoadjuvant paclitaxel, ifosfamide, and cisplatin chemotherapy for metastatic penile cancer: a phase II study. J Clin Oncol. 2010;28(24):3851-7.

107. von der Maase H, Hansen SW, Roberts JT, et al. Gemcitabine and cisplatin versus methotrexate, vinblastine, doxorubicin, and cisplatin in advanced or metastatic bladder cancer: results of a large, randomized, multinational, multicenter, phase III study. J Clin Oncol. 2000;18(17):3068-77.

108. Sternberg CN, de Mulder P, Schornagel JH, et al. Seven year update of an EORTC phase III trial of high-dose intensity M-VAC chemotherapy and G-CSF versus classic M-VAC in advanced urothelial tract tumours. Eur J Cancer. 2006;42(1):50-4.

109. De Santis M, Bellmunt J, Mead G, et al. Randomized phase II/III trial assessing gemcitabine/carboplatin and methotrexate/carboplatin/vinblastine in patients with advanced urothelial cancer who are unfit for cisplatin-based chemotherapy: EORTC study 30986. J Clin Oncol. 2012;30(2):191-9.

110. Perry JR, Rizek P, Cashman R, Morrison M, Morrison T. Temozolomide rechallenge in recurrent malignant glioma by using a continuous temozolomide schedule: the "rescue" approach. Cancer. 2008;113(8):2152-7.

111. Wick W, Puduvalli VK, Chamberlain MC, et al. Phase III study of enzastaurin compared with lomustine in the treatment of recurrent intracranial glioblastoma. J Clin Oncol. 2010;28(7):1168-74.

112. Radford J, Illidge T, Counsell N, et al. Results of a trial of PET-directed therapy for early-stage Hodgkin's lymphoma. N Engl J Med. 2015;372(17):1598-607.

113. Gordon LI, Hong F, Fisher RI, et al. Randomized phase III trial of ABVD versus Stanford V with or without radiation therapy in locally extensive and advanced-stage Hodgkin lymphoma: an intergroup study coordinated by the Eastern Cooperative Oncology Group (E2496). J Clin Oncol. 2013;31(6):684-91.

114. Raemaekers JM, André MP, Federico M, et al. Omitting radiotherapy in early positron emission tomography-negative stage I/II Hodgkin lymphoma is associated with an increased risk of early relapse: Clinical results of the preplanned interim analysis of the randomized EORTC/LYSA/FIL H10 trial. J Clin Oncol. 2014;32(12):1188-94.

115. Pfreundschuh M, Trümper L, Osterborg A, et al. CHOP-like chemotherapy plus rituximab versus CHOP-like chemotherapy alone in young patients with good-prognosis diffuse large-B-cell lymphoma: a randomised controlled trial by the MabThera International Trial (MInT) Group. Lancet Oncol. 2006;7(5):379-91.

116. Fisher RI, Gaynor ER, Dahlberg S, et al. Comparison of a standard regimen (CHOP) with three intensive chemotherapy regimens for advanced non-Hodgkin's lymphoma. N Engl J Med. 1993;328(14):1002-6.

117. Coiffier B, Lepage E, Briere J, et al. CHOP chemotherapy plus rituximab compared with CHOP alone in elderly patients with diffuse large-B-cell lymphoma. N Engl J Med. 2002;346(4):235-42.

118. Habermann TM, Weller EA, Morrison VA, et al. Rituximab-CHOP versus CHOP alone or with maintenance rituximab in older patients with diffuse large B-cell lymphoma. J Clin Oncol. 2006;24(19):3121-7.

119. Halaas JL, Moskowitz CH, Horwitz S, et al. R-CHOP-14 in patients with diffuse large B-cell lymphoma: feasibility and preliminary efficacy. Leuk Lymphoma. 2005;46(4):541-7.

120. Pfreundschuh M, Trümper L, Kloess M, et al. Two-weekly or 3-weekly CHOP chemotherapy with or without etoposide for the treatment of elderly patients with aggressive lymphomas: results of the NHL-B2 trial of the DSHNHL. Blood. 2004;104(3):634-41.

121. Pfreundschuh M, Schubert J, Ziepert M, et al. Six versus eight cycles of bi-weekly CHOP-14 with or without rituximab in elderly patients with aggressive CD20+ B-cell lymphomas: a randomised controlled trial (RICOVER-60). Lancet Oncol. 2008;9(2):105-16.

122. Savage KJ, Chhanabhai M, Gascoyne RD, Connors JM. Characterization of peripheral T-cell lymphomas in a single North American institution by the WHO classification. Ann Oncol. 2004;15(10):1467-75.

123. Lacasce A, Howard O, Lib S, et al. Modified magrath regimens for adults with Burkitt and Burkitt-like lymphomas: preserved efficacy with decreased toxicity. Leuk Lymphoma. 2004;45(4):761-7.

124. Barnes JA, Lacasce AS, Feng Y, et al. Evaluation of the addition of rituximab to CODOX-M/IVAC for Burkitt's lymphoma: a retrospective analysis. Ann Oncol. 2011;22(8):1859-64.

125. Evens AM, Carson KR, Kolesar J, et al. A multicenter phase II study incorporating high-dose rituximab and liposomal doxorubicin into the CODOX-M/IVAC regimen for untreated Burkitt's lymphoma. Ann Oncol. 2013;24(12):3076-81.

126. Pfreundschuh M, Trümper L, Kloess M, et al. Two-weekly or 3-weekly CHOP chemotherapy with or without etoposide for the treatment of young patients with good-prognosis (normal LDH) aggressive lymphomas: results of the NHL-B1 trial of the DSHNHL. Blood. 2004;104(3):626-33.

127. Huguet F, Leguay T, Raffoux E, et al. Pediatric-inspired therapy in adults with Philadelphia chromosome-negative acute lymphoblastic leukemia: the GRAALL-2003 study. J Clin Oncol. 2009;27(6):911-8.

128. Linker C, Damon L, Ries C, Navarro W. Intensified and shortened cyclical chemotherapy for adult acute lymphoblastic leukemia. J Clin Oncol. 2002;20(10):2464-71.

129. Larson RA, Dodge RK, Burns CP, et al. A five-drug remission induction regimen with intensive consolidation for adults with acute lymphoblastic leukemia: cancer and leukemia group B study 8811. Blood. 1995;85(8):2025-37.

130. Rowe JM, Buck G, Burnett AK, et al. Induction therapy for adults with acute lymphoblastic leukemia: results of more than 1500 patients from the international ALL trial: MRC UKALL XII/ECOG E2993. Blood. 2005;106(12):3760-7.

131. Robak T, Dmoszynska A, Solal-Céligny P, et al. Rituximab plus fludarabine and cyclophosphamide prolongs progression-free survival compared with fludarabine and cyclophosphamide alone in previously treated chronic lymphocytic leukemia. J Clin Oncol. 2010;28(10):1756-65.

132. Fernandez HF, Sun Z, Yao X, et al. Anthracycline dose intensification in acute myeloid leukemia. N Engl J Med. 2009;361(13):1249-59.

133. Luskin MR, Lee JW, Fernandez HF, et al. Benefit of high-dose daunorubicin in AML induction extends across cytogenetic and molecular groups. Blood. 2016;127(12):1551-8.

134. Burnett AK, Russell NH, Hills RK, et al. A randomized comparison of daunorubicin 90 mg/m2 vs 60 mg/m2 in AML induction: results from the UK NCRI AML17 trial in 1206 patients. Blood. 2015;125(25):3878-85.

135. Pautas C, Merabet F, Thomas X, et al. Randomized study of intensified anthracycline doses for induction and recombinant interleukin-2 for maintenance in patients with acute myeloid leukemia age 50 to 70 years: results of the ALFA-9801 study. J Clin Oncol. 2010 Feb 10;28(5):808-14.
